# Barriers and Acceptance of Intrauterine Contraceptive Devices (IUCD) Among Married Women of Reproductive Age in Odisha, India

**DOI:** 10.7759/cureus.40919

**Published:** 2023-06-25

**Authors:** Sangita Mukherjee, Dharitri Swain

**Affiliations:** 1 College of Nursing, All India Institute of Medical Sciences, Bhubaneswar, Bhubaneswar, IND; 2 Obstetrics and Gynaecological Nursing, All India Institute of Medical Sciences, Bhubaneswar, Bhubaneswar, IND

**Keywords:** family planning methods, iucds discontinuation, family planning practices, iucds acceptance, contraceptive methods

## Abstract

Introduction: The Copper-T (Cu T-380 A), an intrauterine contraceptive device (IUCD), is widely available and is a highly effective, safe, long-term, and reversible method of contraception. Despite this fact, there is low utilization of IUCDs in India. Hence, this study focused on determining the rate of acceptability of IUCDs, identifying barriers to acceptance of IUCDs, and finding out the cause of IUCD discontinuation.

Methods: A hospital-based cross-sectional study was conducted among 720 married women in the Khordha district, Odisha, India, via a questionnaire and a structured interview schedule. A systematic random sampling method was applied to select the participants. A multivariate logistic regression test was used to determine the factors associated with the acceptance of IUCDs.

Results: This study revealed that only 20.97% of the potential users were currently using IUCDs, 73.75% had never used n IUCD as a contraceptive, and 20.1% of women had discontinued it. Multiple socio-demographic, obstetrical, and family planning behaviours and a lack of awareness were identified to be linked to IUCD acceptability. Fear of adverse effects, family members' objections, availability of other modern contraceptive methods, husband's disagreement, and lack of awareness about the benefits of IUCDs were the most stated reasons for refusal of IUCDs. The most common reason for discontinuing an IUCD was the desire for another child.

Conclusion: The rate of acceptability of IUCDs was quite low in Odisha as compared to other parts of India and, therefore, this study recommends imparting counselling on effective methods of family planning to increase the acceptance of IUCD use.

## Introduction

Estimates from the United Nations indicate that with 1.42 billion people, India will soon become the most populous nation in the world [1]. For a very long time, the Indian government has been quite concerned about population increase. The biggest factor contributing to India's rapid population growth is the unmet need for contraception, which is currently 12.9% higher than it should be, according to the National Family Health Survey (NFHS) 4, 2015-2016 [2]. Because of this, the Government of India's reproductive, maternal, newborn, child, and adolescent (RMNCH+A) approach has placed a greater emphasis on the acceptability of family planning techniques [3].

Numerous family planning approaches are available, but one of the most effective long-acting reversible contraceptive methods is intrauterine contraceptive devices (IUCD), which are implanted inside the uterus to prevent conception. IUCD is the most commonly used contraceptive method in the world but in many developing countries like India, its acceptance and use are minimal though it is freely available in all government settings in India [4]. IUCD usage in India has decreased from 2.0% in NFHS 1 (1992-93) to 1.5% in NFHS 4 (2015-16) [2]. Odisha, a state in eastern India, has a relatively high maternal mortality rate of 136 (Sample Registration System (SRS), 2017-2019) and an infant mortality rate of 38 (SRS-2019) [5,6]. In Odisha, currently, only 1.1% of married women between the ages of 15 and 49 who use contraception really utilize IUCD, per the NFHS 4 [2].

Numerous studies demonstrate that IUCD adoption is being hampered by societal misconceptions about IUCD and a lack of awareness about contraception [7]. The goal of this study was to determine the acceptance rate of IUCD among women in the reproductive age group, the barriers to their acceptance, the frequency of IUCD discontinuation, and the reasons for discontinuation of IUCD. It is important to identify the reasons for the non-acceptance of IUCDs and address the barriers and plans for community-participatory approaches in order to improve the use of IUCDs within the target population.

## Materials and methods

Study participants and study design

This hospital-based descriptive cross-sectional study was conducted from July to December 2021. The study participants were selected from the institutional field practice areas of Khordha districts, Odisha, such as primary health centres (PHCs), community health centres (CHCs), and District Headquarter Hospital, Khordha, purposely as these are the only government health centres in the district providing every modern family planning service. The inclusion criteria were married women of reproductive age between 18-49 years of age attending clinics at selected study centres of Odisha (PHCs Nayapalli, CHC Mendhasal, and District Headquarter Hospital, Khordha district, Odisha). Participation was voluntary with no incentives given for participating in the study. Pregnant women and women or their husbands who had surgical sterilization for permanent family planning methods were excluded from the study. Women with conditions such as distorted uterine anatomy, pregnancy, unexplained vaginal bleeding concerning for pregnancy or pelvic malignancy, pelvic inflammatory disease (PID), postpartum haemorrhage (PPH), etc. were excluded from the study.

The sample size was determined using the single population formula, based on an estimated 57.3% of women in the age group of 15-49 years using any method of contraceptive as a family planning method [2] with the assumption of a 95% confidence interval (CI), 5% margin of error, and 10% nonresponse rate, which yielded the sample size to be 720.

The daily family planning register of the clinic was used to select the sampling frame and a systematic random sampling method was applied to select the participants. Participants who satisfied the inclusion criteria were interviewed by the primary investigator during their free time i.e. before or after consultation with the physician. Based on a secondary data review from October 2018 to October 2021, the clinic had an average of 610 service users per month during the study periods. The first participant was selected by lottery method and then every third patient was picked.

Validity and reliability of the tool

A structured questionnaire tool was used to collect the data, which included three sections: Section-I included socio-demographic data, reproductive history, menstrual history, knowledge towards IUCD and family planning practices. Section-II included dichotomous questions to assess the rate of acceptability of IUCD and barriers to acceptance of IUCD. Section III consisted of open-ended questions to assess the rate of discontinuation of IUCD and the causes of discontinuation of IUCD. The interview method was used to collect the necessary information from the participants.

The content validity of the tool was established by six experts from the field of obstetrics and gynaecology. The overall content validity index of the instrument was appropriate, which shows a high value of Scale-Content Validity Item/Average (S-CVI/Ave=0.97) and the Scale-Content Validity Item/Universal agreement (S-CVI/UN=0.86). The reliability of this tool was obtained by calculating Karl Pearson's coefficient of correlation (r=0.997) and found to be reliable. The tool was converted to a regional language (Odiya) for the understanding of the participants; the Odiya tool was retranslated into English for language validity, done by language experts.

Data collection

The protocol was approved by the Institutional Ethical Committee and formal permission to conduct the study was obtained from the chief district medical officer, Khordha, Odisha. The researcher informed eligible patients about the study and recruited those who agreed to sign the patient consent form. To ensure patient confidentiality, each enrolled patient was assigned a unique patient identification number and interviewed separately. All participants were provided with necessary information about IUCD and future family planning methods at the end of the interview through informational leaflets for further improvement of their knowledge.

Outcome measures

The outcome variables were: the current use of IUCDs and the barriers associated with the acceptance of IUCD as a contraceptive method, which included sociodemographic factors, reproductive factors, and family planning practices. The secondary outcomes were: the rate of discontinuation of IUCD and the causes of discontinuation.

## Results

Socio-demographic and obstetrical characteristics of respondents

A total number of 720 women completed the interview. The mean age of participants was 26.16±5.4 years and their mean age of marriage was 21.34±3.5 years. More than half (52.4%) were in the age group of 25-34 years old and 61.4% of participants and 63.1% of their husband had attained primary education., 57.8% belonged to a low socio-economic group, and family members like mother-in-law/father-in-law/elder brother-in-law/sister-in-law were the decision maker for availing any health service.

More than half of the population (58.6%) were primipara and 60% of them were having one live child at the time of data collection. The majority of women (85.7%) had the youngest child younger than two years and 44.03% of women intended to have more children in the future. Among all participants, 22.5% had a history of abortion, as shown in Table 1.

**Table 1:** Socio-demographic and obstetric characteristics of women of reproductive age group (n=720)

Characteristics	Frequency (f)	Percentage (%)
Parity		
Primipara	422	58.6%
Multipara	298	41.4%
Number of alive child at the time of data collection		
1	432	60%
2	234	32.5%
3	41	5.7%
More than 3	13	1.8%
Preferred space between children		
1 year-2 years	2	0.3%
2 years-3 years	27	3.7%
3 years-4 years	135	18.8%
4 years-5 years	67	9.3%
>5 years	489	67.9%
Space between last two children (n=291)		
Less than 3 years	30	10.3%
3 years or more	261	89.7%
Age of youngest child		
Less than 2 years	617	85.7%
2-5 years	45	6.2%
More than 5 years	58	8.1%
Intention to have more children		
Yes	317	44.03%
No	307	42.64%
Not decided	96	13.33%
Previous pregnancy/pregnancies		
Planned	684	95%
Unplanned	36	5%
Mode of delivery in last pregnancy		
Normal vaginal delivery	489	67.9%
Caesarean Section	231	32.1%
History of abortion/miscarriage		
Nil	558	77.50%
1	141	19.6%
2	17	2.4%
More than 2	4	0.5%

Family planning practices by respondents

It was found that among 720 women, 151 (21%) were currently using IUCD as a contraceptive method; 5.3% of women had used IUCD previously and 65.4% of women did not use any method of contraception previously. Very few participants (0.4%) had used emergency contraceptives in their life. Of these, all have used emergency contraceptive pills (ECPs) as an emergency contraceptive and no one had chosen IUCD as an emergency contraceptive. A total of 436 (60.6%) participants had attended a family planning counseling session regarding IUCD usage; out of them, more than half of the participants (53.2%) had attended the session in their postpartum period. The majority of the subjects stated that husbands (40.7%) as well as family members (46.7%) were against the use of IUCD as a contraceptive method. Health workers were the main source of information about contraceptive methods as stated by 54.3% of subjects.

IUCD use and factors associated with IUCD acceptance

The overall acceptability rate of IUCD among the 720 enrolled participants was 26.25% in their lifetime; while only 20.97% of women currently using IUCD, 73.75% had never used IUCD as a contraceptive and 20.1% of women had discontinued IUCD.

The socio-demographic factors, obstetrical factors, knowledge of IUCD, and personal factors that were significantly associated with IUCD acceptance are presented in Table 2. According to the study, women who were less than 25 years old at the time of marriage (OR=1.788, 99%CI: 1.041-3.07, P=0.006), belonged to Hindu religion (OR=1.627, 99%CI: 1.337-3.347, P<0.001), and low family income (OR=2.23, 99%CI: 1.432-3.475, P<0.001) were most likely to accept IUCD. Obstetrical factors such as primiparous women (OR= 0.486, 99%CI: 0.304-0.778, P<0.001), women whose youngest child was less than two years of age (OR=0.362, 99%CI: 0.163-0.804, P=0.001), and women who intended to have more child in future (OR=0.428, 99%CI: 0.207-0.883, P=0.003) were more likely to accept IUCD as a contraceptive method. More interestingly, those with an abortion history had a five-fold increased likelihood of accepting IUCD (OR=5.204, 99%CI: 3.261-8.303, P 0.001) compared to women without any abortion histories.

**Table 2:** IUCD use and factors associated with IUCD acceptance IUCD: intrauterine contraceptive devices

Sl no	Variables			IUCD acceptability status				OR	99% CI		P value
				Users (n_1_=189)		Non-users n_2_=531			Lower value	Upper value	
				f	%	f	%				
Socio-demographic factors											
1.	Age of woman at the time of marriage		<25years	144	24.2	452	75.8	1.788	1.041	3.07	0.006*
			≥25years	45	36.3	79	63.7				
2.	Religion		Hindu	186	26.7	510	73.3	1.627	1.337	3.347	<0.001*
			Muslim	3	13	20	87				
			Christian	0	0	1	100				
3.	Family income		(₹ per month) ≤10000	82	19.7	335	80.3	2.23	1.432	3.475	<0.001*
			>10000	107	35.3	196	64.7				
Obstetrical factors											
4	Parity		Primipara	134	31.8	288	68.2	0.486	0.304	0.778	<0.001*
			Multipara	55	18.5	243	81.5				
5	Age of youngest child		Less than 2 years	176	28.5	441	71.5	0.362	0.163	0.804	0.001*
			More than 2 years	13	12.6	90	87.4				
6	Intention to have more children		Yes	116	36.6	201	63.4	0.428	0.207	0.883	0.003*
			No	54	17.6	253	82.4				
			Not decided	19	19.8	77	80.2				
7	History of abortion		No	76	15.5	413	84.5	5.204	3.261	8.303	<0.001*
			Yes	113	49	118	51				
Knowledge regarding IUCD											
8	Knowledge score		Good knowledge	166	40	249	60	0.122	0.066	0.226	<0.001*
			Poor knowledge	23	7.5	282	92.5				
Family planning practices											
9		Previously used contraceptive methods	IUCD	38	100	0	0	3.315	3.315	3.315	<0.001*
			Non IUCD users and not using any method	151	22.1	531	77.9				
10		Attended counselling session regarding IUCD	Yes	181	41.6	255	58.4	24.5	9.403	63.77	<0.001*
			No	8	33.3	276	66.7				
11		Time of attending counseling (n=436)	Before delivery	98	66	51	34	0.206	0.118	0.360	<0.001*
			After delivery	83	28.6	204	71.4				
12		Husband’s/ partner’s opinion towards IUCD	Not supporting	14	4.8	279	95.2	0.007	0.003	0.018	<0.001*
			Supporting	163	88.6	21	11.4				
			Lack of knowledge	12	4.9	231	95.1				
13		Family in favor of taking IUCD	Yes	121	86.4	19	13.6	0.028	0.013	0.062	<0.001*
			No	31	39	305	61				
			Lack of knowledge	37	15.2	207	84.8				
14		Source of information about contraceptive methods	Health worker	165	42.2	226	57.8	0.108	0.059	0.198	<0.001*
			Friends and media	24	7.3	305	92.7				

Women who had a good awareness of IUCD had an increased likelihood of using it as a form of contraception (OR=0.122, 99%CI: 0.066-0.226, P<0.001). According to the study's findings, women who had previously used IUCD were 3.315 more likely to accept it (OR=3.315, 99%CI: 3.315-3.315, P<0.001), and those who had received family planning consultation were 24.5 times more likely. It was found that time of counselling session (OR= 0.206, 99%CI: 0.118-0.360, P <0.001), husband's acceptance (OR= 0.007, 99%CI: 0.003-0.018, P <0.001), family members' opinion (OR= 0.028, 99%CI: 0.013-0.062, P <0.001), and source of information about contraceptive methods (OR=0.108, 99%CI: 0.059-0.198, P <0.001) were also associated with the acceptance of IUCD.

Barriers to acceptance of IUCD as contraceptive and discontinuation of IUCDs

The reasons for not accepting the IUCD stated by the non-acceptors revealed that the most common reasons for not accepting IUCD were fear of side effects (38.1%), family members' objection (33.8%), availability of other modern contraceptive methods (30.7%), non-agreement of husband (30.1%), and lack of knowledge/information about IUCD (30%) (Table 3).

**Table 3:** Barriers to acceptance of IUCD as contraceptive and discontinuation of IUCDs IUCD: intrauterine contraceptive devices

Reasons	Yes, n (%)
Fear of side effects on health	274 (38.1%)
Do not want to have a foreign body inside body	127 (17.6%)
Lack of sufficient knowledge/information about IUCD	216 (30%)
Husband not agreeing	217 (30.1%)
Family member’s objection	243 (33.8%)
No need because husband/partner is away	39 (5.4%)
Fear of decreased sexual pleasure	1(0.1%)
Unpleasant experience of friends/family member during IUCD insertion	186 (25.8%)
Planning for permanent sterilization	119 (16.5%)
Unavailability of IUCD in nearby centre	2 (0.3%)
IUCD centre very far	2 (0.3%)
Availability of other modern contraceptive methods	221 (30.7%)
Religious beliefs	17 (2.4%)

Among 189 IUCD acceptors, 38 women (20.1%) were discontinued for various reasons. Most stated causes of discontinuation were the intention of having another child (43.2%), persistent abdominal pain (22.7%), menorrhagia (22.7%), spontaneous expulsion (18.2%), etc. Of the 50% of IUCDs users, 20 (51%) had used IUCD for less than five years, whereas four (10.5%) used it for more than five years, four (10.5%) used it for less than one year, and 11 (29%) used it for less than six months.

## Discussion

Safe and effective contraception is part of quality contraceptive treatment for all women of reproductive age [8]. Copper IUCDs are an efficient technique for limiting fertility as it is a non-hormonal, safe, quickly reversible, low-cost, very successful, long-lasting (Copper T 380A (CuT380A) has a life span of 10 years) method of contraception. Many users find it appealing because of these characteristics [9]. However; their perceived usefulness varies by location [8]. The findings of the present study showed that the rate of acceptability of IUCD is 26.25% among the study participants, which is quite higher than the IUCD acceptability rate found in other parts of India such as central India (11.98%) and south India (19.72%) [7,10]. In this study, 20.97% of women were active users, compared to 39% of users in a study done in North India (Faridabad Hariyana) [11], and this percentage of current users in the present study is also lesser than the findings of studies conducted in various parts of Ethiopia [12-14]. While the current study revealed a higher percentage of current users than earlier studies in Bolangir, Odisha, India (17.5%), western Uttar Pradesh, India (14.4%), and Bhaktapur, Nepal (7%) [15-17], these variations in the current use of IUCD may be due to study settings, knowledge about IUCD, level of awareness, and source of information.

Belief in myths and misconceptions about family planning has been positively associated with contraceptive discontinuation and subsequent unmet need [18]. As a result, people are much more aware of its issues or problems than of its benefits. Among the non-acceptors of IUCD, 38.1% of women mentioned fear of the negative impact on health, 30% of them reported insufficient knowledge/information, 30.1% replied spouse did not agree, and 17.6% of them did not want to have a foreign body inside their body. These issues are almost equivalent to the findings of a previous study conducted in Nepal by Khatri et al. [17].where 48.25% of participants who were unwilling to use IUCD mentioned the concern of adverse health effects, 31.6% said they lacked knowledge, and 23.5% indicated their husband's opposition. Another study in Nepal's Kathmandu valley found that 30%, 31.8%, and 6.4% were reluctant to adopt IUCD due to fear of harmful impacts, inadequate knowledge, and objection from their husbands, respectively [19]. A treatment centre-based cross-sectional study in Southeast Ethiopia reported that 24.8% of women refused to use IUCD due to concerns about complications, and 17.7% rejected it due to husband rejection [20], similar to the findings of the current study. Fear of problems was cited by 69.96% of women in a study done by Sharma et al. as the reason for their refusal of IUCD [11]. A study conducted in Bolangir, Odisha, reported that 25.77% of women were afraid of pain and excessive bleeding, 42.96% told that they needed to take advice from husbands, and a major (66.94%) reason for not accepting was inadequate knowledge about IUCD [15]. Hence, it is noticeable that while in a previous study in Odisha, 66.94% stated insufficient knowledge as the major reason, this was reduced to 30% in the present study. This noticeable change may be because of various awareness approaches from the government and family planning counselling during prenatal or antenatal visits.

Thus, from the findings of the report of 2014 and the current study, we may presume the importance of providing eligible couples with pertinent information and counselling on the use, safety, adverse reactions, and dangers of IUCDs so that they may make better choices about which form of contraception is right for them.

Gonie et al. reported in their study that 19.8% of participants rejected IUCD use as a contraceptive because of religious beliefs whereas in this present study, only 2.4% of women mentioned religious belief as their reason for rejecting IUCD [20]. Mishra et al. reported in their project that 0.04% stated religious beliefs as the reason behind not accepting IUCD in Odisha [15], which strongly coordinates with the present study. The contrary finding between the present study and the study by Gonie et al. might be due to different locations or the difference in the number of women from different religions in this study.

Regarding discontinuation of IUCD, an earlier study documented that 9.35% of females used IUCD for more than five years, 53.8% used it for one to five years, 9.35% used it for six months to one year and 27.48% of women decided to remove within six months of insertion [9]. These findings are quite analogous to the present study findings. These early removals of IUCD may be the effect of gaining knowledge/information from relatives and neighbours rather than health workers, which creates a fear factor inside them regarding IUCD. Hence, post-insertion counselling for follow-up and confirming that IUCD is inside the cavity of the uterus by ultra-sonography can reassure women and motivate them to keep the device in place.

Strengths and limitations of the study

The strengths of this study were in the use of a pre-validated set of structured questionnaires and face-to-face interviews conducted by trained interviewers. It was conducted in community settings, which increases the scope of generalization of study findings. Large numbers of women were recruited from multiple field practice areas involving both urban and rural communities, which increases the strength of the present study by enlarging the scope to generalize the findings to the whole population. The study addressed all possible reasons for not accepting IUCD and the causes of removing IUCD by previous IUCD users making the present study more informative.

The first limitation of this study was that we did not include any private health centres in this study; participants were chosen from the government community health centres, which may not be generalizable to all health centres. Although sufficient samples from both urban and rural communities were collected, they were not distributed equally, which may have limited the ability to address the study's findings about the acceptance rate of contraception among rural and urban women. Due to time restrictions, the existing IUCD acceptors were not followed up on in this study.

Relevance of the findings: implications for clinicians and policy-makers/healthcare providers

This study shows a high unmet need for contraception despite easy access and available subsidies for the groups at the highest risk for unintended pregnancy. Women express that contraceptive effectiveness is the most important quality of a contraceptive, but method-specific knowledge on IUCD effectiveness is lacking and a high proportion of women use less effective methods. Hence, continuous contraceptive counselling is important so that they can actively choose and not be at risk of passive continued use of a method that may not suit them the best. Along with this, post-insertion counselling regarding warning signs that indicate the need to return to the health centre, follow-up counselling, and pre-removal counselling should be provided to users, which will reduce complaints of side effects and promote adherence. Increasing knowledge of fertility and awareness of contraceptive effectiveness is necessary. Promoting the use of IUCDs with maintained acceptability and increased satisfaction with bleeding patterns are possible ways forward in the effort to reduce the rates of unintended pregnancy.

## Conclusions

Contraception has a vital role in reducing unplanned pregnancies, unsafe abortions, and associated mortalities. However, IUCD acceptance rate was found to be poor in this investigation. During contraceptive counseling, method effectiveness and the additional health benefits of IUCDs should be emphasized. Additionally, actions are required to reach individuals with low usage of effective contraception as well as those who never proactively seek contraception.
